# Ipsilateral Concomitant Intertrochanteric and Femoral Shaft Fractures Associated With Metatarsal Bone Fractures: An Unusual Presentation

**DOI:** 10.7759/cureus.32032

**Published:** 2022-11-29

**Authors:** Ahmed A Ismail, Abdulkrim S Alzahrani, Majed A Alanazi, Aishah M Mousa, Dania T Shaikh

**Affiliations:** 1 Orthopedic Surgery, King Fahad General Hospital, Jeddah, SAU; 2 Critical Care, Qurayat General Hospital, Qurayat, SAU

**Keywords:** fixation, metatarsal bone fracture, fracture reduction, ipsilateral intertrochanteric and femoral shaft fractures, intertrochanteric fractures

## Abstract

Concomitant ipsilateral intertrochanteric and femoral shaft fractures with metatarsal bone fractures in young adults are extremely rare, and there are only a few reports regarding these fractures in the literature. High-energy trauma is a well-known attributable factor for this type of fracture. In this report, we presented a rare case of ipsilateral intertrochanteric and femoral shaft fractures with metatarsal bone (MTB) fractures in a 42-year-old male patient who was a victim of a road traffic accident (RTA). He was managed by closed fracture reduction and fixation with gamma nails, lag and locking screws, and K-wires. This particular combination of fractures is scarce. Reporting such a case to orthopedic surgeons is useful for management since these injuries present technical and infrastructural challenges, especially in resource-limited hospitals.

## Introduction

Intertrochanteric fractures are defined as extracapsular fractures of the proximal femur that occur between the greater and lesser trochanters and are classified based on stability [1]. Femoral shaft fracture is defined as any fracture between 5 cm distal to the lesser trochanter and 5 cm proximal to the adductor tubercle according to the AO Foundation/Orthopedic Trauma Association (AO/OTA) classification system, a well-established system for classifying femoral shaft fractures [2]. It is unusual to see a case of concurrent ipsilateral intertrochanteric and femoral shaft fracture since only 1-9% of all femoral shaft fractures were found to have simultaneous proximal femur fractures and a quarter of them are intertrochanteric fractures [3]. The mechanism of injury for this type of fracture is often due to high-energy trauma, such as road traffic accidents (RTA) and falling from heights. As a result, patients may suffer from multiple injuries. Surgical fixation of these multiple fractures while maintaining a satisfactory outcome is challenging and associated with a high rate of complications [4,5]. We present a young patient with right intertrochanteric and femoral shaft fracture associated with right second and third metatarsal bone (MTB) fracture from RTA.

## Case presentation

A 42-year-old male patient, without any previous medical or surgical conditions, was transferred from the Khulais hospital to the King Fahad General Hospital emergency department in Jeddah for right ipsilateral intertrochanteric and femur shaft fractures. The patient had been involved in a road traffic accident, and he was wearing a seat belt during the accident. The patient did not get ejected from the car, which didn’t roll over.

Upon examination at the King Fahad General Hospital emergency department, the patient was alert, conscious, oriented, and vitally stable. The right lower limb was on skin traction that was removed for better inspection. He had lacerations on the lateral aspect of the right thigh, multiple abrasions around the right knee, and a 2x3 cm wound on the mid-lateral aspect of the right leg, sutured with four stitches. There was tenderness over the right thigh, leg, and foot. He was moving his foot, but the range of motion of the right knee and hip was limited due to pain. Pulses were palpable, and distal nerves were intact. 

**Figure 1:**
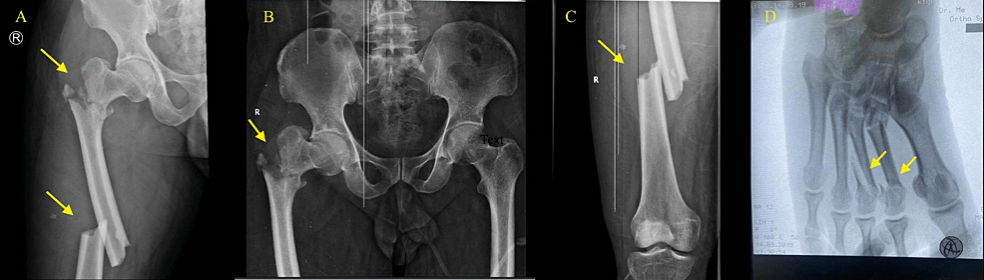
Right intertrochanteric fracture with ipsilateral femur shaft fracture (A, B, and C); right second and third metatarsal bone fractures (D)

**Figure 2:**
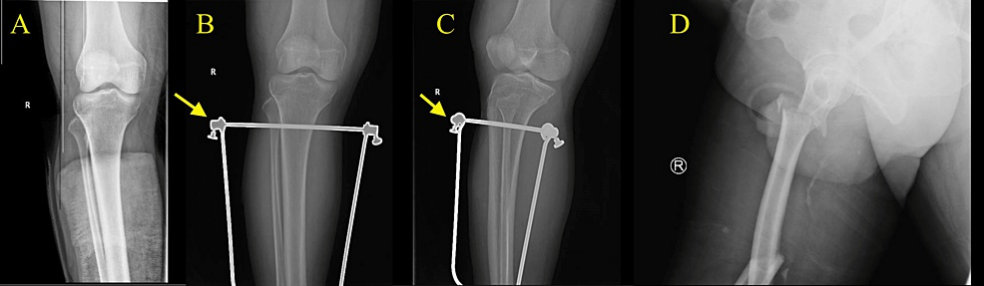
Before applying proximal tibia skeletal traction (A) and after traction image (B, C, and D)

**Figure 3:**
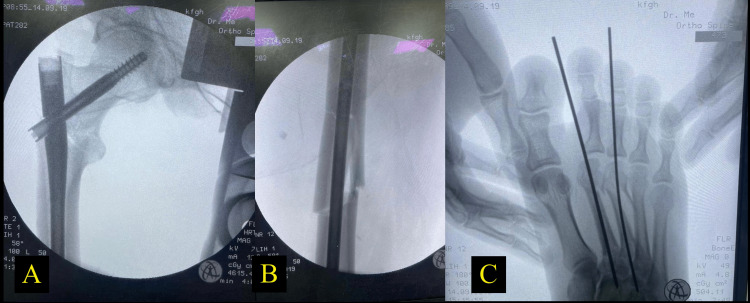
Closed reduction with intramedullary nail for the intertrochanteric fracture (A) and femur shaft fracture (B) Closed reduction with K-wires for the right second and third metatarsal bone fractures (C)

**Figure 4:**
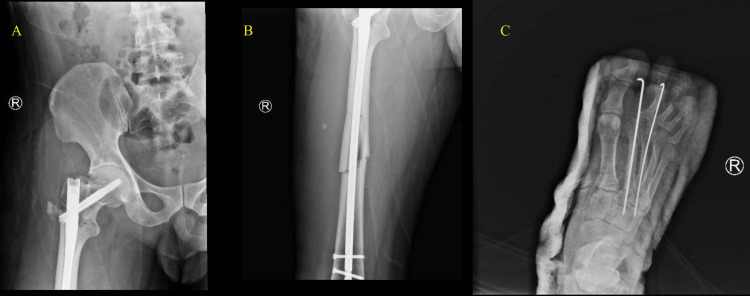
Immediate postoperative X-ray for the femur nail (A, B) and metatarsal K-wires (C)

**Figure 5:**
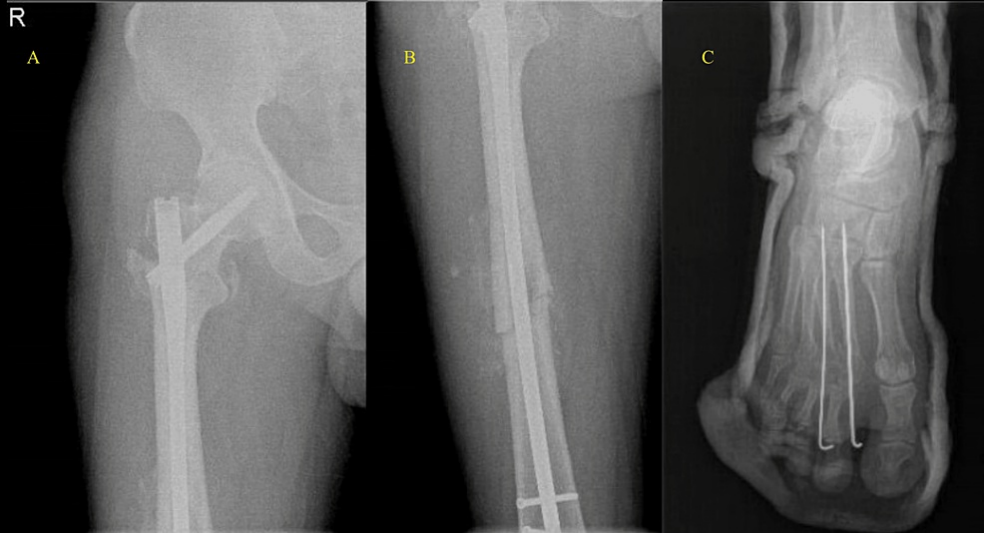
Two weeks postoperative X-rays for the femur fractures (A, B) and metatarsal fractures (C)

X-ray images showed a right intertrochanteric fracture with ipsilateral femur shaft fracture and the right second and third MTB fractures (Figure 1). The patient was administered analgesics and prophylactic anticoagulant (enoxaparin), and proximal tibia skeletal traction was applied (Figure 2) after closed fracture reduction. During the surgical operation, a gamma nail size 10*40mm x 125 degrees was used to fix the femur fractures. A lag screw size 95mm and two distal locking screws static size 45mm were used to fix the nail. A closed reduction with K wire fixation of the right second and third MTB was also done (Figure 3), and a below-knee back slab was applied. The patient was shifted to the recovery area in good condition without any complications. Post-operative X-rays showed all fixatives in the correct places (Figure 4). Post-operative analgesia and broad-spectrum antibiotic (cefuroxime) were given, and prophylactic enoxaparin started eight hours postoperatively. On day one after surgery, physiotherapy therapy sessions began with non-weight bearing. The patient was discharged with no complications on analgesia and prophylactic enoxaparin on the fourth day postoperatively. An outpatient follow-up appointment was given. Two weeks later in the clinic, the patient had a regular follow-up visit without complications, with no signs of infection at the operation sites, and physiotherapy was done as scheduled. Moreover, an X-ray was done to compare it with future X-rays regarding bone healing (Figure 5).

## Discussion

Intertrochanteric fractures mostly occur in older people, usually secondary to minor osteoporosis-related trauma. Young people rarely sustain this type of fracture, mainly due to high-energy trauma, such as RTA, as in our case.

Intertrochanteric fractures occurring concomitantly with femoral shaft and metatarsal bone fractures are even rarer. A retrospective study analyzing 34 cases of ipsilateral hip and femoral shaft fractures reported from 1995 to 2005 found that more than three-quarters of these fractures were from RTA [6]. Of all cases in this study, 20 were extracapsular, and 14 were intracapsular. Most (76.5%) recovered successfully during an average 28-month follow-up. Another study involving 65 German patients with ipsilateral proximal and shaft femur fractures reported from 2004 to 2013 showed that the all-in-one device technique, the “rendezvous” technique, and the conventional technique were used to treat 36, 9, and 16 patients, respectively. Two years later, 93.4% had completely healed, and four died of multiorgan failures [4]. A review of 14 cases of ipsilateral proximal and shaft femoral fractures treated with combined fixation (bridge-link) from 2012 to 2016 showed that one proximal femoral fracture and one femoral shaft fracture had nonunion at 13 months and 12 months postoperatively, respectively [7]. 

Though during high-velocity traumas, such as RTA, the force is transmitted along the femoral shaft, causing impaction and leading to shaft fracture. The force then progresses to the proximal femur, resulting in basicervical femur fractures [4]. However, intertrochanteric fractures previously treated, then refractured through the femoral neck, have been reported, and local osteoporosis due to fixation devices, inadequate fixation, and imperfect reduction have been hypothesized to be the causes [5,8].

Our case is of high interest due to the combination of intertrochanteric and femoral shaft fracture associated with metatarsal bone fracture, which was never previously reported to date. Most reported cases involved the femur at different levels and didn’t involve the metatarsal bone fractures. Moreover, our patient was relatively younger and less likely to have osteoporosis than most patients previously reported who commonly get these types of femur fractures with osteoporosis as a risk factor. Since the patient is relatively younger, the surgeon should prioritize the preservation of the femoral head during surgery to fix intertrochanteric fractures. Gamma nails stabilized by lag and locking screws, as used in our case, help achieve bone union while the femur head is preserved.

In this case of fracture complex, it is advised to fix the femoral shaft first in order to achieve better leg control when fixing more complex and unstable fractures of the proximal femur or femur head [7,9]. Though there are various techniques to treat these types of femoral fracture complexes, there is no data on which one is the best. However, most surgeons prefer lag screw fixation and reamed intramedullary nailing for the femoral neck and shaft fractures, respectively [10,11]. Since union takes a long time for femoral fractures, nonunion should be diagnosed after six to eight months post-fixation without union [11].

K-wire fixation of MTB fractures is simple and easy and can be done as an open or closed fracture reduction technique. In addition, it minimizes tissue damage, pain, and discomfort caused by malposition after MTB fractures [12].

## Conclusions

This case report showed that intertrochanteric and femoral shaft fractures might occur even in association with MTB fractures in young people without osteoporosis despite being very rare. Since this type of fracture complex is rare, surgeons should always prioritize preserving the femoral head in young patients and choose appropriate techniques to achieve it and allow restoration of function and minimize complications.
